# Association between cigarette smoking and suicide in psychiatric inpatients

**DOI:** 10.1186/1617-9625-11-5

**Published:** 2013-02-18

**Authors:** Sharifi Hooman, Hessami Zahra, Mitra Safa, Farhadi Mohammad Hassan, Masjedi Mohammad Reza

**Affiliations:** 1 Tobacco Prevention and Control Research Center (TPCRC), Masih Daneshvari Hospital, Shaheed Beheshti Medical Science University, Shaheed Bahonar Ave, Darabad, Tehran, IRAN; 2 National Research Institute of Tuberculosis and Lung Diseases (NRITLD), Masih Daneshvari Hospital, Shaheed Beheshti Medical Science University, Tehran, Iran; 3 Department of psychology, Masih Daneshvari Hospital, Shaheed Beheshti Medical Science University, Tehran, IRAN; 4 Drug Abuse Research Center, University of social welfare and rehabilitation sciences, Tehran, Iran

**Keywords:** Nicotine, Cigarette, Suicide, Iran

## Abstract

**Introduction:**

Cigarette smoking is the single largest preventable cause of death and disability in the industrialized world and it causes at least 85% of lung cancers, chronic bronchitis and emphysema. In addition smokers are at a higher risk from psychiatric co-morbid illness such as depression and completed suicide.

**Methods:**

We conducted a cross-sectional survey in which we targeted all patients with serious mental illness (SMI) who were admitted in Razi mental health Hospital in Tehran, Iran. We recruited 984 participants, who were receiving services from Razi mental health Hospital and hospitalized for at least two days between 21 July to 21 September, 2010. Nine hundred and fifty patients out of this figure were able to participate in our study.

**Results:**

The final study sample (n = 950) consisted of 73.2% males and 26.8% females. The mean age was 45.31 (SD=13.7). A majority of participants (70%) was smoker. A history of never smoking was present for 25.2% of the study sample; while 4.8% qualified as former smokers and 70.0% as occasional or current smokers. Two hundred and nineteen participants had attempted suicide amongst them 102 (46.6%) once, 37 (16.9%) twice, and 80 (36.5%) attempted more than two times in their life time. In regression model, gender, age, and cigarette consumption were associated with previous suicide attempts and entered the model in this order as significant predictors.

**Conclusion:**

There is an association of cigarette smoking and suicide attempt in psychiatric inpatients. Current smoking, a simple clinical assessment, should trigger greater attention by clinicians to potential suicidality and become part of a comprehensive assessment of suicide risk.

## Introduction

Cigarette smoking is the single largest preventable cause of death and disability in the industrialized world [1]. More than 430,000 deaths per year in the US alone are attributed to smoking [1]. Tobacco smoking is the cause of at least 85% of lung cancers, chronic bronchitis and emphysema [2]. In addition smokers are at a higher risk from psychiatric co-morbid illness such as depression and completed suicide [3-5]. Besides, suicide is one of the preventable public health problems. Although the mechanism by which smoking may increase the risk of suicidal behavior is not clear, several studies report statistically significant integrated associations between smoking and suicide [3-5].

Psychiatric patients have markedly elevated rates of nicotine dependence [6]. Beyond the adverse health implications of smoking, according to some studies bipolar patients who smoke might be at higher risk of suicidal behavior and suicide attempts, independent of substance abuse and anxiety disorder co morbidity [7]. Along with severity of depression and having made a prior suicide attempt, smoking was a robust predictor of suicidal behavior following a major depressive episode in bipolar disorder, even after controlling for other factors [8] and regardless of gender [9]. Additionally, a study of adolescents with bipolar disorder found that cigarette smoking was independently associated with suicide attempts and substance use disorders [10,11].

Age, gender, alcohol abuse/ dependence, and affective illness are a few of the identified risk factors for suicidal ideation, suicide attempts and suicide [12-14] and also appear to be correlated with smoking [4,5,12,14]. As well, studies have found a significant association between depressive disorders, anxiety symptoms, and alcohol abuse/dependence with smoking [5,12,14].

Considering that mentioned studies found associations between a few psychiatric problems and suicide, we are to control hospitalized patients for potential confounders, that is, features shared by smokers and by persons who commit suicide, such as income, race, and history of serious physical illness and alcohol abuse.

It would be important to assess whether there is a relationship between suicidal attempts and smoking and if there is any this may add to the body of data that speaks to uncountable benefits of quitting smoking as well as identify a window of opportunity for suicide prevention. The current study examines the relationship between smoking history and the incidence of suicide attempts. We hypothesize that current and former smokers will be more likely to report suicide attempts compared to never smokers.

## Method

We conducted a cross-sectional survey in which we targeted all patients with serious mental illness (SMI) who were admitted in Razi mental health Hospital in Tehran, Iran. The vast majority of patients with a diagnosis of SMI, in this city, are admitted in this referral hospital. Services include medication management, individual and group therapy, rehabilitation, and education. Many clients receive additional support in the form of rehabilitation programming or housing from Rehabilitation and Well-fare Organization.

### Sample

We sought to obtain a representative sample of people with SMI receiving community mental health services. We recruited 984 participants, who were receiving services from Razi mental health Hospital at least two days between 21 July to 21 September, 2010. These participants were either new administered or patients who had been hospitalized since previous days or weeks. Nine hundred and fifty patients out of this figure were able to participate in our study. All study participants were hospitalized in this hospital, their insight level was more than one, and were able to communicate and be understood in Persian.

### Procedures

Razi mental health Hospital has about 10 sectors. The research staff visited each sector of this hospital, provided information about the study, answered questions, and negotiated strategies to access participants. A research assistant recruited participants at the mental health team offices during regular operating hours. The research staff explained the study in detail, obtained written, fully informed consent, and administered the questionnaire [15].

### Ethical approval

Ethical approval was obtained from the Behavioral Research Ethics Board of Tobacco Prevention and Control Research Center of Shaheed Beheshti University of Medical Science. Approval to conduct the research was obtained from the University of Welfare and Rehabilitation Sciences.

### Measures

The questionnaire, which included several scales and items, requiring 20–45 minutes to complete, was administered by the research staff including three main fields,

#### Demographics

The demographic items included: age, gender, marital status, current living situation, housing type, financial support, and social assistance/welfare.

#### Suicide attempts

All participants were asked about previous suicide attempts with the following details: times, methods, and the time of committing suicide. The main question was “In your entire lifetime did you ever attempt suicide?” Responses were coded as yes or no.

#### Insight

According to *Comprehensive Textbook of Psychiatry* (2000) a system adopted by Kaplan and Sadock was used to evaluate patients’ insight level.

#### Tobacco use patterns

Smoking status was determined by asking the participants if they had "ever" smoked, whether they had smoked more than 100 cigarettes in their lifetime, when they smoked their last cigarette, and if they smoked every day [16]. The participants were classified as non-smokers (had never smoked or smoked less than 100 cigarettes), ex-smokers (had smoked more than 100 cigarettes, but had not smoked in the past 30 days), or current smokers (had smoked more than 100 cigarettes and had smoked in the past 30 days).

Nicotine dependence was measured with the Fagerström Test for Nicotine Dependence (FTND) [17]. This test is appropriate for the assessment of nicotine dependence in smokers with schizophrenia [18]. The coding algorithm yields a total score of 0-6(simplified FTND). Scores above 3 are indicative of a high level of dependence.

#### Psychiatric diagnosis

All diagnoses in the summary for hospitalizations are made and finalized by a board-certified attending psychiatrist. For all of the participants we were permitted to access their medical records. Diagnosis was based on DSM-IV-TR formulation collected from the summary of a patient’s first admission during the study period.

### Analysis

A total of 950 people participated in the study, which represents approximately 96.5% of the clients who received care from mental health teams in Razi hospital. The data from these clients were cleaned and screened before analysis to ensure missing data were random in occurrence and that all data were within their expected ranges. Descriptive analysis of the sample (N = 950) employed chi square tests to determine the associations between study variables. Independent sample *t*-tests employing Levine’s test for equality of variance were employed to examine the relationships between psychiatric diagnosis and the continuous variables. First, we employed univariate logistic regression analyses to identify the study variables associated with suicide attempt and conducted these analyses for the entire sample. In the second step, variables that were associated with suicide attempt at *p* ≤ .25 were included in the multivariate logistic regression models (all participants). To obtain the most parsimonious and stable models, we then trimmed them by removing statistically non-significant variables sequentially by examining the Wald statistic and comparison of the likelihood ratios. If the likelihood ratio test was significant when a non-significant variable was removed (i.e., *p* < .05), then the variable was added back to the model. Once the main effects models were finalized, all possible interactions between diagnostic category and the other variables were examined. All analyses were conducted with IBM SPSS Statistics 16.

## Results

### Characteristics of the subjects

The final study sample (n = 950) consisted of 73.2% males and 26.8% females. The mean age was 45.31 (SD=13.7). 63.3% of the subjects were single, 15.3% of them were married and rest of them were divorced, widow or separated. The majority of the study population (57.6%) were unemployed and had lower education than high school (79.3%). Only 14.8% of subjects own personal house. 67.9% of the study population was under supervision of the head of the family or well-fare organization (Table 1).

**Table 1:** **Socio**-**demographic characteristics of the participants at baseline**

**Variables**	**Numbers**	**Percentages**
Sex (male)	695	73.2
Males age (year)	43.3±12.7*	_
Females age (year)	50.7±14.4*	_
Education		
Illiterate and primary	248	48.8
Guidance and high school	273	31.1
Diploma and upper	177	20.1
Social class		
Clerical or Non-manual skilled	42	4.6
Manual skilled	180	19.7
Unskilled or semiskilled	728	75.7
Tenure		
Owner occupied	119	14.8
Rented/other	861	85.2
Marital status		
Married	144	15.3
Single	596	63.3
Widowed, divorced or others	210	21.4

* Mean ± Standard deviation.

### Smoking pattern and nicotine dependency of subjects

A majority of participants (70%) was smoker. A history of never smoking (i.e. tobacco, water-pipe) was present for 25.2% of the study sample; while 4.8% qualified as former smokers and 70.0% as occasional or current smokers. The mean age of initiating smoking was 20.1 ± 6.2 years old, the mean number of the daily smoked cigarettes was 14 ± 10.11 cigarettes for smoker subjects. According to FTND the intensity of Fagerstrom test in 64.4% of subjects was high (Table 2).

**Table 2:** **Participant**’**s smoking status**, **habits and dependence at baseline**

	**Number (percent)**	**Male**	**Female**
Participants Status			
Smoker	571 (70%)	512 (78.4%)	59 (36.2%)
Ex-smoker	39 (4.8%)	27 (4.1%)	12 (7.4)
Non-smoker	206 (25.2%)	114 (17.5%)	92 (56.4)
Total	816 (100%)	653 (100%)	163 (100%)
Nicotine Dependency			
Low (less or equal to 3)	181 (31.7%)	167 (32.6%)	14 (23.7%)
High (more than 3)	368 (64.4%)	331 (64.6%)	37 (62.7%)
No reply	22 (3.9%)	14 (2.7%)	8 (13.6%)
Total	571 (100%)	512 (100%)	59 (100%)

### Characteristics of mental illness

At baseline, 28.2%, of the study sample met diagnostic criteria for neuro-cognitive disorders, 85.2% psychotic disorders, 42.1% mood disorders, 12% anxiety disorders, 6.9% substance abuse/dependence disorders, 36% personality disorders, 11.2% eating disorders, 23.9% sleep disorders, and 5.5% sexual disorders. 98.5% of the study sample had at least one comorbid disorder.

### Suicide attempts

Two hundred and nineteen participants had attempted suicide, 23% of the total sample, amongst them 102 (46.6%) once, 37 (16.9%) twice, and 80 (36.5%) attempted more than two times in their life time. Univariate analysis showed an association between suicide attempt and the variables of age, level of education, marital status, accommodation status, employment status, type of psychiatric disorder, cigarette smoking, and alcohol abuse in men and women (Table 3).

**Table 3:** Factors related to suicide attempt in men and women using univariate logistic regression

** Variable**	**Univariate analysis for women**	**P-value**	**Univariate analysis for men**	**P-value**
	**OR (95% CI)**		**OR (95% CI)**	
Age	0.95 (0.93-0.98)	0.001	0.94 (0.93-0.96)	<0.001
Level of education				
Less than high school	Reference		Reference	
High school	2.83 (1.06-7.56)	0.038	1.39 (0.89-2.18)	0.143
University	3.64 (0.82-16.01)	0.088	2.25 (1.04-4.85)	0.038
Marital status				
Single	Reference		Reference	
Married	1.92 (0.66-5.57)	0.229	1.58 (1.01-2.48)	0.045
Divorced, separated, and other	2.26 (1.08-4.76)	0.030	1.30 (0.80-2.11)	0.287
Tenure				
Private	4.82 (2.18-10.67)	<0.001	4.43 (2.54-7.71)	<0.001
Rented	8.36 (1.93-36.22)	0.004	5.88 (3.05-11.32)	<0.001
Other	Reference		Reference	
Unemployment				
Yes	Reference		Reference	
No	5.43 (2.51-11.82)	<0.001	1.46 (1.03-2.06)	0.033
Type of psychiatric disorder				
Schizophrenia	0.25 (0.10-0.62)	0.003	1.00 (0.65-1.55)	0.971
Bipolar	0.28 (0.10-0.79)	0.016	1.64 (0.97-2.77)	0.061
Depression	0 (0–0)	0.999	1.10 (0.64-1.88)	0.715
Other	Reference		Reference	
Cigarette smoking^†^				
Yes	2.24 (1.05-4.80)	0.037	2.09 (1.29-3.41)	<0.001
No	Reference		Reference	
Alcohol abuse				
Yes	21.78 (2.37-200.31)	0.006	3.91 (2.61-5.86)	<0.001
No	Reference		Reference	

† Current or ex-smoker versus never smoker.

## Discussion

It is noteworthy that almost two third of the study participants were current smokers; this is more than four times the 2007 smoking rate of 14.8% in Iran reported by Centre for Disease Control and Management [19]. Thanks to our large sample size, our study was able to describe smoking patterns in psychiatric diagnostic groups. Our data was not same as previous work done by Ziaaddini et al. in a different inpatient setting and geographic region in Iran [20]. Average smoking rates in their sample for psychiatric patients was, 51.6% [20] versus 70% in our sample. Although, a counter-argument can also be made that studies which are community-based and have a large sample size may be more representative, but generally it has proved that smoking rate among psychiatric inpatient is higher than general population.

Regarding the low proportion of females in this study, it seems that psychiatric inpatients’ gender has the same ratio in different studies in different provinces. Ziaaddini et al. showed that a proportion of 2/1 in male/female held true in two groups, schizophrenia and other psychiatric disorders in their study [20].

Generally, the outcomes of our study corroborate the hypothesis of a link between tobacco smoking and suicide attempts. The findings also support http://Bronisch and colleagues’ report [21] of increased the risk for onset of suicide attempts in smokers compared to never smokers aged 14 to 24, and our study extends this relationship to hospitalized individuals up to age 84.

It has been proposed that the relationship between smoking and suicide attempts was possibly a result of depressive disorder and/or anxiety symptoms as well as alcohol dependence [14]. Our study has shown that although alcohol abuse/dependence and neuro-cognitive disorders have had a significant relationship with suicide attempts, but there were no significant relationship with suicide attempts and mood disorders. A review of research showed that on average, 40% of suicide attempters and 37% of individuals who died by suicide had acute alcohol use [21]. Given Substance abuse/dependence disorders, the prevalence of alcohol abuse in our study population was 1.3%. Although it seems to be less important, but we should consider that drinking alcohol is forbidden by religious rules in Iran, the likelihood of underreporting in this category must be taken into account.

Limitations of the study are that we have no data on completed suicide and that the number of suicide attempts was small. To analyze these rare outcomes, a much larger sample and longer follow-up period are needed. Moreover, the results of this study cannot be applied or generalized any other group of population.

## Conclusion

There is an association of cigarette smoking and suicide attempt in psychiatric inpatients. Current smoking, a simple clinical assessment, should trigger greater attention by clinicians to potential suicidality and become part of a comprehensive assessment of suicide risk. While it is unknown whether reducing nicotine dependence in psychiatric patients would impact suicidality, these results indicate that further research is needed to better understand the link between cigarette smoking and elevated suicidality in psychiatric inpatients, with especial attention to alcohol abuse/dependence and some psychiatric disorders like Schizophrenia, Bipolar, and Depression.

Furthermore, considering the low percentage of former smokers amongst psychiatric population which resulted in high probability of smoking initiation after smoking experience, there is a clear need for specially tailored interventions for both smoking cessation and lowering the risk of suicide for psychiatric patients and in the future more resources should be targeted to solve these health concerns.

## Competing interests

The authors declare that they have no competing interests.

## Authors’ contributions

SH carried out the smoking section of study and participated in study design and draft of manuscript. HZ participated in the design of the study and performed the statistical analysis, MS and FM carried out psychological section, MM participated in its design and coordination and helped to draft the manuscript. All authors read and approved the final manuscript.

## References

[B1] Rockville MD How Tobacco Smoke Causes Disease: The Biology and Behavioral Basis for Smoking-Attributable Disease A Report A Report of the Surgeon General Executive Summary 2010 21452462

[B2] Salma K Tobacco Control and Prevention, A guide for low income countries 1998 Paris: IUATLD 12 70

[B3] Doll R Peto R Mortality in relation to smoking, 20 years’ observations on male British doctors BMJ 1976 2 6051 1525 1536 10.1136/bmj.2.6051.1525 1009386 PMC1690096

[B4] Iwasaki M Akechi T Uchitomi Y Tsugane S Cigarette smoking and completed suicide among middle-aged men, a population-based cohort study in Japan Ann Epidemiol 2005 15 4 286 292 10.1016/j.annepidem.2004.08.011 15780776

[B5] Riala K Viilo K Hakko H Rasanen P STUDY-Seventy Research Group. Heavy daily smoking among under 18-year-old psychiatric inpatients is associated with increased risk for suicide attempts Eur Psychiatry 2007 22 4 219 222 10.1016/j.eurpsy.2006.06.001 17127036

[B6] Grant BF Hasin DS Chou SP Stinson FS Dawson DA Nicotine dependence and psychiatric disorders in the United States: results from the national epidemiologic survey on alcohol and related conditions Arch Gen Psychiatry 2004 61 1107 1115 10.1001/archpsyc.61.11.1107 15520358

[B7] Ostacher MJ Nierenberg AA Perlis RH The relationship between smoking and suicidal behavior, comorbidity, and course of illness in bipolar disorder J Clin Psychiatry 2006 67 1907 1911 10.4088/JCP.v67n1210 17194268

[B8] Oquendo MA Galfalvy H Russo S Prospective study of clinical predictors of suicidal acts after a major depressive episode in patients with major depressive disorder or bipolar disorder Am J Psychiatry 2004 161 1433 1441 10.1176/appi.ajp.161.8.1433 15285970

[B9] Oquendo MA Bongiovi-Garcia ME Galfalvy H Sex differences in clinical predictors of suicidal acts after major depression: a prospective study Am J Psychiatry 2007 164 134 141 10.1176/appi.ajp.164.1.134 17202555 PMC3785095

[B10] Wilens TE Biederman J Adamson JJ Further evidence of an association between adolescent bipolar disorder with smoking and substance use disorders: a controlled study Drug Alcohol Depend 2008 95 188 198 10.1016/j.drugalcdep.2007.12.016 18343050 PMC2365461

[B11] Goldstein BI Birmaher B Axelson DA Significance of cigarette smoking among youths with bipolar disorder Am J Addict 2008 17 364 371 10.1080/10550490802266151 18770078 PMC2905883

[B12] Makikyro TH Hakko HH Timonen MJ Smoking and suicidality among adolescent psychiatric patients J Adolesc Health 2004 34 3 250 253 14967350 10.1016/j.jadohealth.2003.06.008

[B13] Bromet EJ Havenaar JM Tintle N Kostyuchenko S Kotov R Gluzman S Suicide ideation, plans and attempts in Ukraine, findings from the Ukraine World Mental Health Survey Psychol Med 2007 37 6 807 819 10.1017/S0033291707009981 17288636

[B14] Bronisch T Hofler M Lieb R Smoking predicts suicidality, Findings from a prospective community study J Affective Disorders 2008 108 1–2 135 145 10.1016/j.jad.2007.10.010 18023879

[B15] Roberts LW Roberts B Psychiatric research ethics: an overview of evolving guidelines and current ethical dilemmas in the study of mental illness Biol Psychiatry 1999 46 8 1025 1038 10.1016/S0006-3223(99)00205-X 10536739

[B16] Srinivasan TN Thara R Smoking in schizophrenia – all is not biological Schizophr Res 2002 56 1–2 67 74 12084421 10.1016/s0920-9964(01)00187-6

[B17] Forchuk C Norman R Malla A Martin ML McLean T Cheng S Diaz K McIntosh E Rickwood A Vos S Gibney C Schizophrenia and the motivation for smoking Perspect Psychiatr Care 2002 38 2 41 49 12132630 10.1111/j.1744-6163.2002.tb00656.x

[B18] Weinberger AH Reutenauer EL Allen TM Termine A Vessicchio JC Sacco KA Easton CJ McKee SA George TP Reliability of the Fagerstrom Test for Nicotine Dependence, Minnesota Nicotine Withdrawal Scale, and Tiffany Questionnaire for Smoking Urges in smokers with and without schizophrenia Drug Alcohol Depend 2007 86 2–3 278 282 16876968 10.1016/j.drugalcdep.2006.06.005

[B19] Meysamie A Ghaletaki R Haghazali M Asgari F Rashidi A Khalilzadeh O Esteghamati A Abbasi M Pattern of tobacco use among the Iranian adult population: results of the national Survey of Risk Factors of Non-Communicable Diseases (SuRFNCD-2007) Tob Control 2010 19 2 125 128 10.1136/tc.2009.030759 20008159 PMC2989156

[B20] Ziaaddini H Kheradmand A Vahabi M Prevalence of Cigarette Smoking in Schizophrenic Patients Compared to Other Hospital Admitted Psychiatric Patients Addiction and Health 2009 1 1 38 43 PMC3905500 24494081

[B21] Cherpitel CJ Borges GL Wilcox HC Acute alcohol use and suicidal behavior: a review of the literature Alcohol Clin Exp Res 2004 28 18S 28S 15166633 10.1097/01.alc.0000127411.61634.14

